# New indices of arterial stiffness measured with an upper‐arm oscillometric device in active versus inactive women

**DOI:** 10.14814/phy2.13574

**Published:** 2018-02-27

**Authors:** Ryota Kobayashi, Soichiro Iwanuma, Nobuyuki Ohashi, Takeo Hashiguchi

**Affiliations:** ^1^ Center for Fundamental Education Teikyo University of Science Tokyo Japan; ^2^ Department of School Education Teikyo University of Science Tokyo Japan

**Keywords:** Arterial pressure‐volume index, arterial velocity pulse index, cross‐sectional study, regular aerobic exercise

## Abstract

Arterial velocity pulse index (AVI) and arterial pressure‐volume index (API), new indicators of arterial stiffness, are risk factors for the development of cardiovascular disease. Regular aerobic exercise decreases arterial stiffness. In fact, pulse wave velocity (PWV), index of arterial stiffness, is lower in endurance‐trained than in untrained young adults. However, the effect of regular aerobic exercise on AVI and API remains unknown. This study investigates the effect of regular aerobic exercise on AVI and API, new indicators of arterial stiffness. We gathered data from 18 recreationally active females (active group, age: 18 ± 1 years, 2 ± 2 h/week, 3 ± 2 times/week, ≥2 years of aerobic endurance training) and 18 recreationally inactive females (inactive group, age: 18 ± 1 years, ≥2 years without such training) in a cross‐sectional study. Height, body weight, body mass index, AVI, API, brachial blood pressure, heart rate, and 20‐m multistage shuttle run test were measured in a quiet room at a temperature between 24°C and 25°C. AVI and API were lower in the active group than in the inactive group (*P* < 0.01). Number of 20‐m shuttles was negatively correlated with AVI (*P* < 0.01, *r* = −0.8) and API (*P* < 0.01, *r* = −0.8). These results suggest that regular aerobic exercise training decreases AVI and API in young females.

## Introduction

Increased arterial stiffness is an independent risk factor for the development of cardiovascular disease (Blacher et al. 1999). Pulse wave velocity (PWV) and cardio‐ankle vascular index (CAVI) are now used to assess arterial stiffness (Tomiyama and Yamashina 2010). Although these techniques are clinically and experimentally accepted, they have some disadvantages: (1) a long measurement time, (2) a physical load (with pressure from a blood pressure [BP] cuff on both arms and ankles or from squeezing a tonometry sensor into the carotid artery), and (3) requirement of knowledge and skill in applying tonometry transducers (Okamoto et al. 2016). Therefore, another method might be of value/use to check arterial stiffness periodically.

Recently, arterial velocity pulse index (AVI) and arterial pressure‐volume index (API), two novel methods for evaluating systemic and peripheral arterial stiffness, respectively, have been gaining attention. Because AVI and API can be calculated by simply applying an oscillometric cuff to the upper left arm for 2 min, they can be measured more easily than PWV and CAVI. Moreover, AVI and API are correlated with PWV and CAVI (Komine et al. 2012; Okamoto et al. 2016; Ueda et al. 2016; Komatsu et al. 2017; Zhang et al. 2017). For instance, Zhang et al. (2017) showed that AVI and API correlated with systemic (brachial‐ankle) PWV in a study of 183 individuals. Moreover, increases in AVI and API are risk factors for the development of cardiovascular disease (Okamoto et al. 2016; Sasaki‐Nakashima et al. 2017). Sasaki‐Nakashima et al. (2017) reported that API was showed to be independently related to both Framingham cardiovascular risk score and the Suita score, suggesting that API is a useful predictor of future cardiovascular events. Therefore, it is therefore necessary to clarify ways in which AVI and API can be maintained and improved to reduce cardiovascular disease risk.

Regular aerobic exercise decreases arterial stiffness. Otsuki et al. (2007) reported that aortic PWV is lower in endurance‐trained (≥2 years of aerobic endurance training) than in untrained (≥2 years of no aerobic endurance training) healthy young men. In addition, Vlachopoulos et al. (2010) found that aortic (Carotid‐femoral) PWV was significantly lower in the marathon runners compared with the age‐matched controls. Thus, AVI and API might be lower in active subjects (regular aerobic endurance training) compared with inactive subjects. However, the effect of active on AVI and API has not been elucidated. This study investigates for the first time the new indices of arterial stiffness, AVI and API, in active and inactive subjects. We hypothesized that AVI and API would be lower in active than in inactive age‐matched individuals.

## Materials and Methods

### Subjects

Thirty‐six healthy young females were assigned to a group of either recreationally active (active group, age: 18 ± 1 years, *n* = 18) or inactive (inactive group, age: 18 ± 1 years, *n* = 18). Participants in the active had an active lifestyle (2 ± 2 h/week, 3 ± 2 times/week, ≥2 years of low‐ and moderate‐intensity aerobic endurance training [jogging and walking]; assessed by the International Physical Activity Questionnaire [IPAQ]), and participants in the inactive group had a sedentary lifestyle (≥2 years without such training). All participants were studied during the early follicular phase of the menstrual cycle to avoid any hormonal influences on arterial stiffness. None of the female participants was taking oral contraceptives. All participants were normotensive (<140/90 mmHg) nonsmokers without symptoms or a history of chronic diseases (Table 1). All were fully informed about the experimental procedures as well as the purpose of the study before providing written consent to participate. The Ethics Committee at Teikyo University of Science approved this study, which proceeded in accordance with the guidelines for human experimentation published by the university's Institutional Review Board (17033). This study also confirmed to the principles defined in the Declaration of Helsinki.

**Table 1:** Participant characteristics

	Active group (*n* = 18)	Inactive group (*n* = 18)
Age (years)	018 ± 1	18 ± 1
Height (cm)	159 ± 5	159 ± 6
Weight (kg)	54 ± 9	54 ± 7
BMI (kg/m^2^)	21 ± 3	21 ± 2
Number of 20‐m shuttles	56 ± 4^a^	31 ± 6
Predicted *V*O_2_ max (mL/kg per min)	39 ± 1^a^	33 ± 1

Values are mean ± SD. BMI, body mass index; *V*O_2_ max, maximal oxygen consumption; SD, standard deviation.

a *P *<* *0.01 versus in active group.

### Study design and sample size

**Figure 1:**
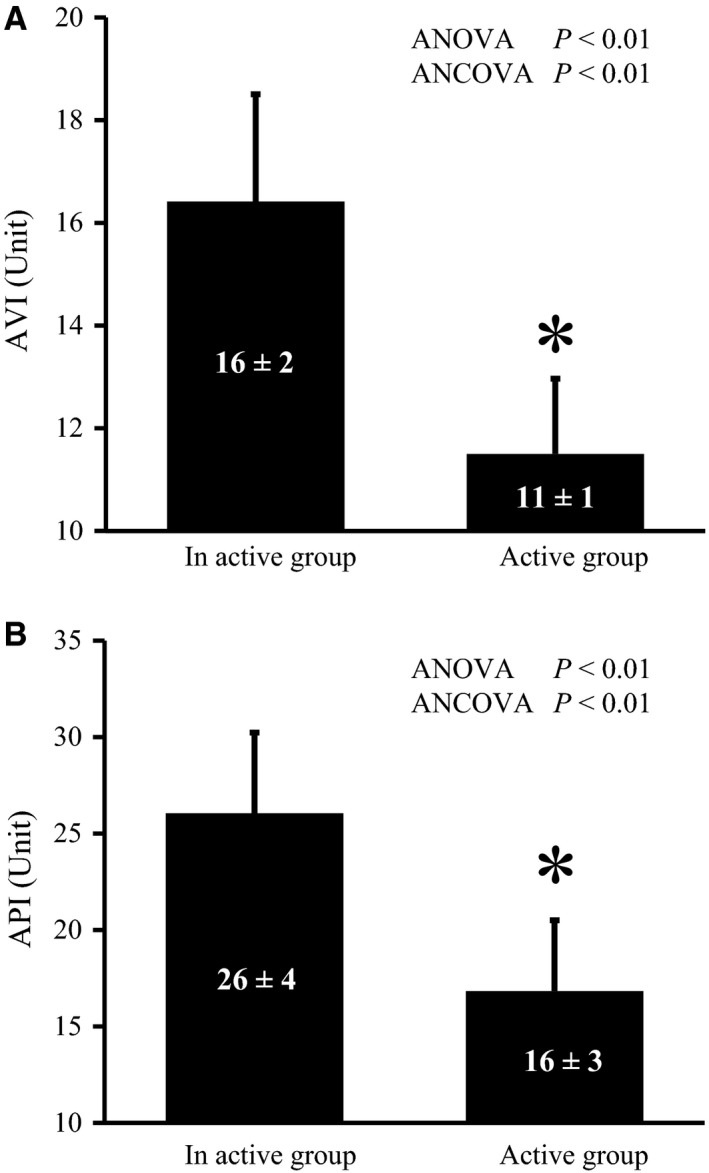
AVI (A) and API (B) in both groups. Values are mean ± SD. *P <* *0.01 versus in active group. AVI, arterial velocity pulse index; API, arterial pressure‐volume index; SD, standard deviation.

After coming to the laboratory, IPAQ, height, body weight, and body mass index (BMI) were measured. After 5 min of seating rest, AVI, API, brachial BP, heart rate (HR) were measured. Thereafter, 20‐m multistage shuttle run test (MSRT) was conducted (Fig. 1). All participants presented at least 4 h after eating and did not consume alcohol, caffeine, or participate in exercise for 24 h before starting the tests. All parameters were measured at a constant room temperature (24–25°C) in a quiet room.

Before starting the study, we performed power analyses using G*Power 3 (Faul et al. 2007) to determine the appropriate sample size. To detect a significant AVI and API with training, using a 10 and 18 standard deviation (SD) in active group and 15 and 26 SD in inactive group (based on data from the first five participants), a power of 0.8 and an alpha of 0.05, a sample of six inactive and six active females was needed.

### Anthropometrics

Height and weight were measured to the nearest 0.5 cm using a stadiometer, and body composition was determined using the WB‐150 PMA Body Composition Analyzer (TANITA Co. Ltd., Tokyo, Japan).

### Arterial velocity pulse index and arterial pressure‐volume index

AVI and API were measured as follows (Sueta et al. 2015a,b; Okamoto et al. 2016). AVI and API were obtained using a time series of occlusive cuff pressure and amplitudes of pulse oscillations. AVI and API reflect systemic and peripheral arterial stiffness, respectively. Left brachial BP and brachial arterial pulse waves were simultaneously measured using a form PASESA AVE‐1500 (Shisei Datum, Tokyo, Japan). Left brachial arterial pressure waveforms were stored by oscillometric sensors attached to the left common brachial arteries. Until now, PWV was evaluated according to the blood vessel in the long‐axis direction, but AVI and API were measured with new principle. In the AVI measurement, the peak amplitude ratio of the amplitude of differential waveform was evaluated according to the cuff pressure. In the API measurement, the arterial volume was evaluated according to the short‐axis direction of the blood vessel. The principles and formulas for AVI and API were previously reported by Komine et al. (2012) and Sueta et al. (2015a). The indices were measured using cuff oscillometry with PASESA AVE‐1500 (Shisei Datum) by trained researcher (one person). The patients were in a sitting position during the measurement. The validity and reliability of AVI and API measured using this method have been confirmed by previous reports (Sasaki‐Nakashima et al. 2017). The normal distribution of AVI and API was confirmed using Kolmogorov–Smirnov tests.

The daily coefficients of variation (CVs) at the laboratory were 4.9 ± 0.5% and 5.6 ± 1.1% for AVI and API, respectively. Moreover, within‐day CVs at the laboratory were 5.3 ± 0.5% and 5.7 ± 1.1% for AVI and API, respectively.

### Blood pressure and heart rate

Brachial systolic blood pressure (SBP), mean arterial pressure (MAP), diastolic blood pressure (DBP), pulse pressure (PP), and HR at rest while sitting were measured using an automated oscillometric device from PASESA AVE‐1500 (Shisei Datum) over the left brachial arteries.

The daily CVs at the laboratory were 4.0 ± 3.5%, 4.6 ± 8.2%, 5.0 ± 3.0%, 1.8 ± 5.6%, and 11.0 ± 5.5% for SBP, MAP, DBP, PP, and HR, respectively.

### 20‐m MSRT

On the test field, lines were marked on the floor 20 m apart, with a turning area beyond each of the two lines. An audiotape with recorded audio signals for the test was played through a sound system in the field (Léger and Lambert 1982). The test was conducted according to previously reported standards (Léger and Lambert 1982) and, because 20‐m MSRT results are correlated with maximal oxygen uptake (*V*O_2_ max) (Paradisis et al. 2014), estimated *V*O_2_ max could be determined for each participant. Moreover, within‐day CVs at the laboratory were 5.3 ± 0.5% and 5.7 ± 1.1% for AVI and API, respectively. The validity and reliability of 20‐m MSRT measured using this method have been confirmed by previous reports (Léger and Lambert 1982).

The daily CVs at the laboratory were 2.1 ± 0.3% and 9.4 ± 1.3% for 20‐m MSRT and *V*O_2_ max.

### Statistical analyses

All data are presented as mean ± SD. The normal distribution of all data was confirmed using Kolmogorov–Smirnov tests. Between‐group means comparisons were performed with independent *t* tests. Correlations among AVI, API, and SBP were examined using the Pearson product moment correlation coefficient. Analysis of covariance (ANCOVA) included SBP and HR as a covariate. All analyses were performed using JMP^®^ Pro, version 12 (SAS Institute Inc., Tokyo, Japan). Differences were considered significant at *P* < 0.05.

## Results

### Participant characteristics

Table 1 shows participant characteristics of both groups. Age, height, weight, and BMI did not differ between groups. The 20‐m shuttles (total count) and predicted *V*O_2_ max were significantly higher in the active group than in the inactive group (*P* < 0.01).

### AVI and API in both groups

Figure 1 shows AVI and API in both groups. AVI was significantly lower in the active group than in the inactive group (*P* < 0.01, Fig. 1A). API was significantly lower in the active group than in the inactive group (*P* < 0.01, Fig. 1B). These results remained the same after normalizing AVI and API for SBP and HR when analyzed by ANCOVA.

### BP and HR in both groups

Table 2 shows BP and HR in both groups. SBP, MBP, DBP, PP, and HR were significantly lower in the active group than in the inactive group (*P* < 0.01).

**Table 2:** Blood pressure and HR in both groups

	Active group (*n* = 18)	Inactive group (*n* = 18)
Brachial SBP (mmHg)	104 ± 6^a^	120 ± 8
Brachial MAP (mmHg)	81 ± 4^a^	90 ± 6
Brachial DBP (mmHg)	71 ± 5^a^	75 ± 7
Brachial PP (mmHg)	34 ± 7^a^	44 ± 7
HR (beats/min)	67 ± 5^a^	73 ± 6

Values are mean ± SD. HR, heart rate; SBP, brachial systolic blood pressure; MAP, mean arterial pressure; DBP, diastolic blood pressure; PP, pulse pressure; SD, standard deviation.

a *P *<* *0.01 versus in active group.

### Correlation between AVI, API, SBP, and PP in both groups

**Figure 2:**
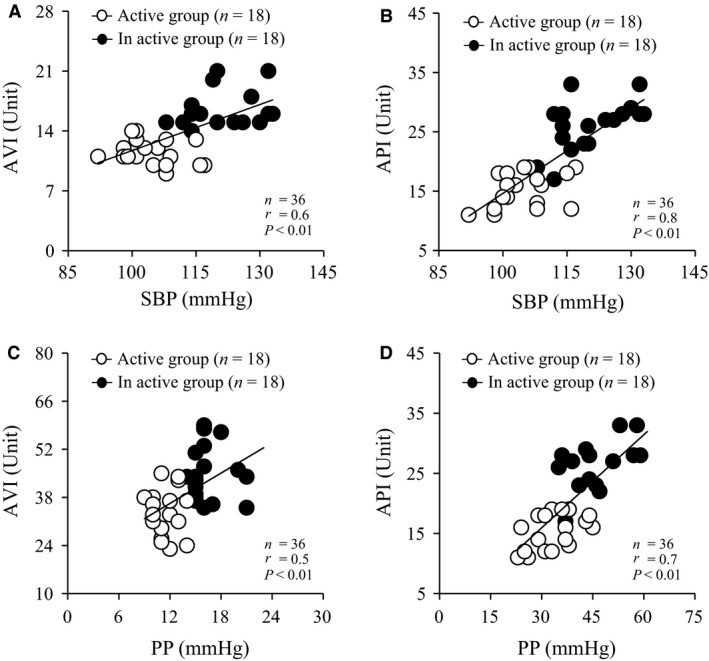
Correlation between SBP and AVI (A) and API (B) and PP and AVI (C) and API (D) in both groups. Values are mean ± SD. AVI, arterial velocity pulse index; API, arterial pressure‐volume index; SBP, systolic pressure; PP, pulse pressure; SD, standard deviation.

Figure 2 shows the correlation between AVI, API, SBP, and PP in both groups. SBP was positively correlated with AVI (*P* < 0.01, *r* = 0.6, Fig. 2A) and API (*P* < 0.01, *r* = 0.8, Fig. 2B). PP was positively correlated with AVI (*P* < 0.01, *r* = 0.5, Fig. 2C) and API (*P* < 0.01, *r* = 0.7, Fig. 2D).

### Correlation between AVI, API, number of 20‐m shuttles, and predicted *V*O_2_ max in both groups

**Figure 3:**
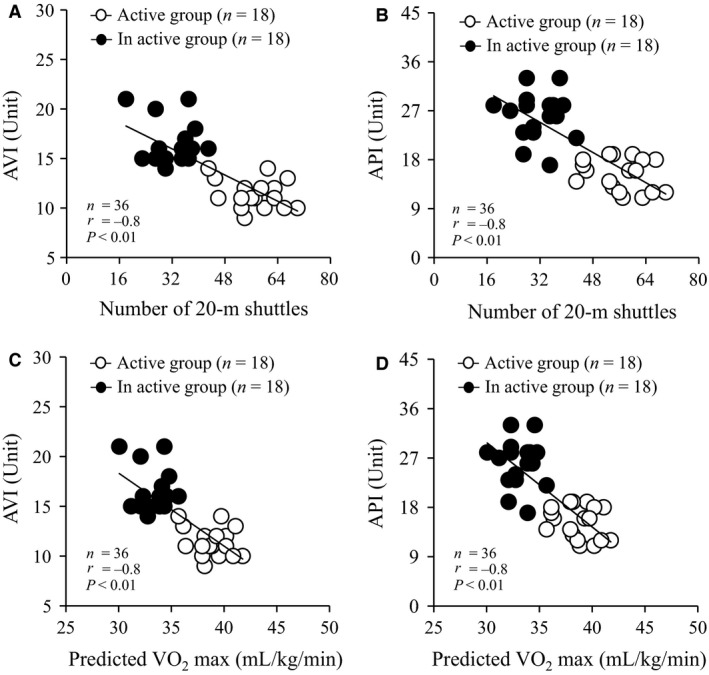
Correlation between number of 20‐m shuttles and AVI (A) and API (B) and predicted VO_2_ max and AVI (C) and API (D) in both groups. Values are mean ± SD. AVI, arterial velocity pulse index; API, arterial pressure‐volume index; VO_2_ max, maximal oxygen uptake; SD, standard deviation.

Figure 3 shows the correlation between AVI, API, number of 20‐m shuttles, and predicted *V*O_2_ max in both groups. Number of 20‐m shuttles was negatively correlated with AVI (*P* < 0.01, *r* = −0.8, Fig. 3A) and API (*P* < 0.01, *r* = −0.8, Fig. 3B). Predicted *V*O_2_ max was negatively correlated with AVI (*P* < 0.01, *r* = −0.8, Fig. 3C) and API (*P* < 0.01, *r* = −0.8, Fig. 3D).

## Discussion

The novel finding of this study is that AVI and API were lower in the active group than in the inactive group in females. These results suggest that regular aerobic exercise training in females decreases AVI and API, and for health management, it might be necessary to measure AVI and API in addition to BP measurement by oscillometric device.

Although the effect of aerobic exercise on arterial stiffness has been investigated before, we were examining for the first time the effect of aerobic exercise on two new indices (AVI and API) on arterial stiffness. Otsuki et al. (2007) reported lower aortic arterial stiffness in endurance‐trained (≥2 years of aerobic endurance training) than in untrained healthy young men (≥2 years of no aerobic endurance training). Tanaka et al. (2000) found that arterial compliance is lower in exercisers than in nonexercisers. We found that AVI and API were lower in the active group than in the inactive group. Moreover, *V*O_2_ max values correlate with aortic PWV across a wide age range (Vaitkevicius et al. 1993). We found that predicted *V*O_2_ max was negatively correlated with AVI and API. Thus, regular aerobic exercise might decrease AVI and API in healthy humans.

The present study was not designed to examine possible mechanisms by which AVI and API were reduced in the active group, but we propose the following explanations. API is calculated using cuff pressure and arterial volume (Komine et al. 2012). Komine et al. (2012) reported that the slope of the curve between brachial cuff pressure and arterial volume varies with arterial compliance. Thus, the changes in API are affected by arterial compliance. Changes in arterial compliance are associated with arterial SBP (Benetos et al. 1997). We showed that API and brachial arterial SBP were lower in the active group than in the inactive group and correlated with API and brachial arterial SBP. Otsuki et al. (2007) showed that arterial compliance in young people was high in endurance‐trained compared with untrained healthy young men. Therefore, a low API in the active group might be associated with decreases in brachial arterial SBP that are due to increases in brachial arterial compliance. However, we did not measure brachial arterial compliance, which is a limitation of the present study. AVI is influenced by changes in SBP of the peripheral arteries (e.g., brachial arteries) (Zhang et al. 2017). Vlachopoulos et al. (2010) showed that brachial SBP and aortic (carotid‐femoral) PWV in young people were low in active compared with inactive peers. We found that AVI and brachial arterial SBP were lower in the active group than in the inactive group and correlated with AVI and brachial arterial SBP. Therefore, the present study's finding of lower AVI in the active group than in the inactive group could be associated with decreased reflected wave from decreased brachial arterial SBP. However, we did not measure reflected wave, which is a limitation of the present study. On the other hand, changes in arterial stiffness are influenced by vascular endothelial function (Wilkinson et al. 2004). Vascular endothelial cells play an important role in the regulation of vascular activity by producing vasoactive substances such as nitric oxide (NO, vasodilator) and endothelin‐1 (ET‐1, vasoconstrictor) that participate in the regulation of arterial stiffness (Wilkinson et al. 2004). Kearney et al. (2014) showed that regular moderate aerobic exercise (30 min/day; 5 days/week) decreases arterial stiffness and increases the release of NO. Otsuki et al. (2007) reported that aortic PWV and ET‐1 are lower in endurance‐trained than in untrained healthy young men. Therefore, the finding of lower AVI and API in the active group than in the inactive group might be associated with a change in endothelial function. However, we did not measure vascular endothelial function, which is a limitation of the present study.

Reduced arterial stiffness associated with regular aerobic exercise is a benefit of cardiovascular health. In fact, although aerobic exercise is widely recommended to prevent arterial stiffness (Kawakami et al. 2013), the absence of a simple method of confirming the effect of aerobic exercise on arterial stiffness may be an obstacle to regular exercise. In the present study, in the active group, API and AVI, which can be easily measured, were lower than in the inactive group. Therefore, the results of this study might be useful for health consciousness and health maintenance, and might be used to motivate individuals to start or continue regular exercise. Medical expenses in Japan are increasing (Sasaki et al. 2015), and health maintenance (e.g., prevention of arterial stiffness) should be implemented from a young age. In fact, it has been reported that the risk of cardiovascular disease will decrease after 20 years if a healthy lifestyle (exercise habits, healthy diet, no smoking, or smoking cessation) is maintained from a young age (Liu et al. 2012). Measurement of AVI and API is less burdensome for the examinee, and the arterial stiffness can be confirmed in 2 min. Thus, the results of this study might be useful for grasping arterial stiffness by regular aerobic exercise. Therefore, it might be important to measure the blood vessel index simultaneously with the sitting BP just like the conventional BP monitor.

The present study had some limitations. The study was limited to healthy young females, so our results cannot simply be extrapolated to other populations. Age is associated with an enhanced cardiovascular risk as well as arterial stiffness (Braunwald and Zipes 2005). The study population was 18 years old with BMI 21; the findings with other populations might therefore differ particularly in elderly patients who have increased prevalence of cardiovascular risk factors. We did not measure arterial compliance, reflected wave, vascular endothelial function, gold standard *V*O_2_ max test, or female hormones, which could have important effects on arterial stiffness. In future research, the mechanism (e.g., arterial compliance) of the effect of aerobic exercise on AVI and API should be examined. The sample size was relatively small; however, the magnitude of the effect on arterial stiffness was adequate and similar to those in previous studies of aerobic exercise effects (cross‐section study) on arterial stiffness (Otsuki et al. 2007, 2008).

In conclusion, AVI and API were lower in the active group than in the inactive group in females. These results suggest that regular aerobic exercise training in females decreases AVI and API. Moreover, the results of the present study suggest that AVI and API must provide more prognostic information than simply assessing SBP and DBP.

## Conflict of Interest

The authors declare that they have no conflict of interest relevant to the content of this manuscript.
